# Spotlight on deep carbon research

**DOI:** 10.1038/s41467-019-12747-9

**Published:** 2019-10-22

## Abstract

Professor Marie Edmonds is a volcanologist at the University of Cambridge. She is interested in the role of magmatic volatiles in magma genesis, volcanic eruptions, and volatile geochemical cycling. Dr. Robert Hazen is a geologist at Carnegie Science and executive director of the Deep Carbon Observatory. His latest research has focused on the co-evolution of the geospheres and biospheres, and mineral diversity and distribution. Marie and Robert apply their research to help understand the chemical and biological roles of carbon in Earth.

**Figure:**
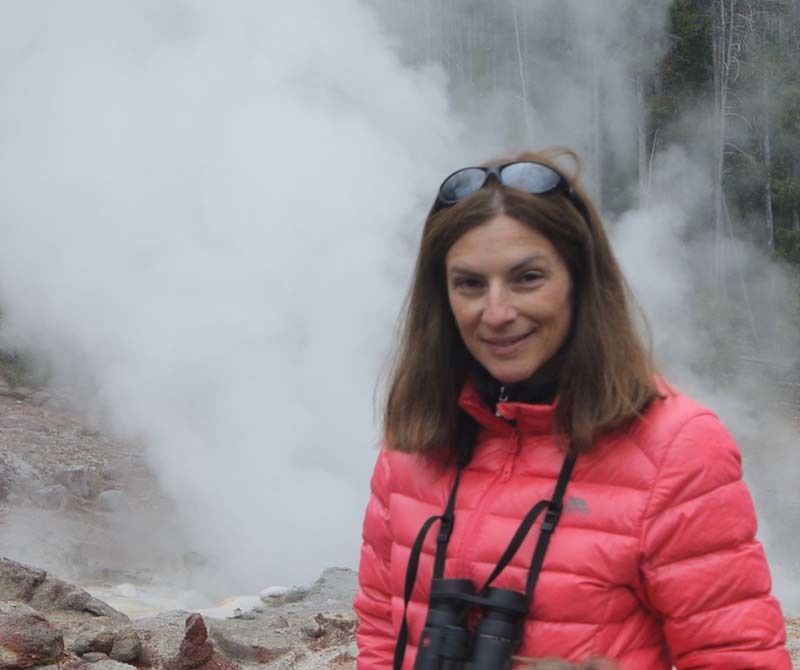
Marie Edmonds

Could you describe what deep carbon science is and why it is important to research?

Carbon plays unique roles in our dynamic and evolving planet. The sixth element provides the chemical foundation for life, it continues to serve as the primary source of our energy needs, it inspires a host of remarkable new materials, and it plays a disproportionate role in Earth’s uncertain, changeable climate and environment. These multi-faceted aspects of carbon have inspired decades of intensive research, most of which focuses on components of the near-surface carbon cycle—the oceans, atmosphere, and biosphere—that display rapid changes and that are most influenced by human activities.

Deep carbon research takes a more encompassing view by considering the estimated 90% of Earth’s carbon that is hidden from view in the planet’s interior. We explore the forms, quantities, movements, and origins of carbon sequestered in Earth’s inaccessible core, cycling in the deep mantle, reacting in deep fluids, and lurking in a fascinating subsurface biosphere. We cannot understand carbon in Earth—we cannot place the changeable surface world in context—without the necessary baseline provided by deep carbon research.

**Figure:**
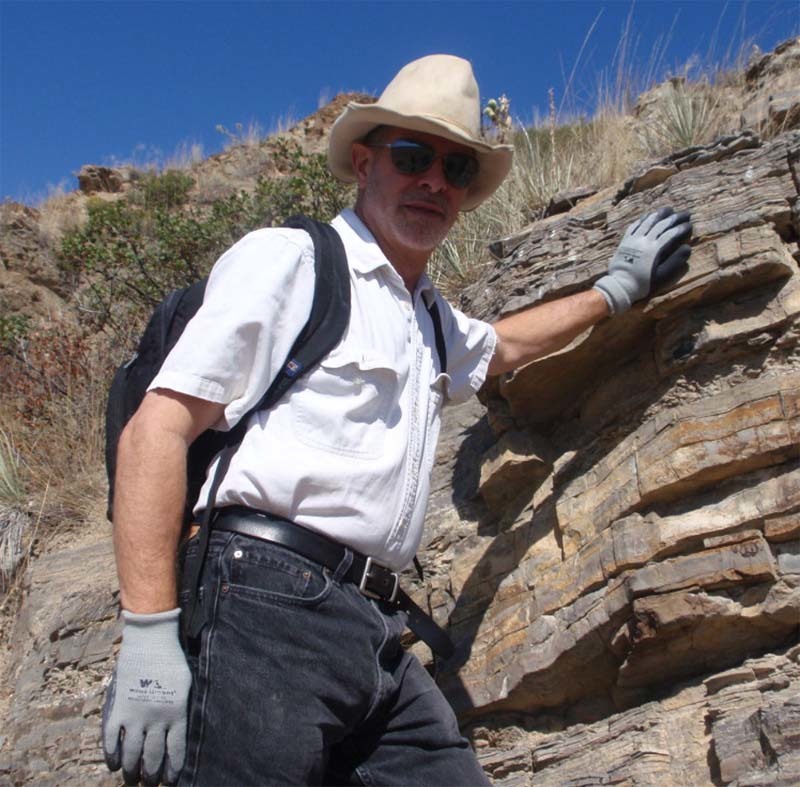
Robert Hazen

Could you highlight the major breakthroughs across deep carbon research in the past ten years?

Deep carbon science has produced transformative discoveries in a range of fields over the past decade. We now understand much better where in Earth carbon is stored, and in what forms. The iron-rich core, for example, may hold more than two-thirds of the total carbon in Earth in the form of iron carbide species. The mantle too holds a plethora of carbon forms, in both solid and fluid states, including high-pressure forms of carbonate minerals that may persist to great depth, transported there by subducting oceanic slabs rich in carbonate sediments produced by marine organisms. A revolutionary new model, the Deep Earth Water Model (DEW), allows understanding of the nature and reactivity of fluids in the deep mantle. Inclusions in “super-deep” diamonds have revealed for the first time in situ evidence of lower mantle mineralogy, including calcium silicate perovskite and carbon-rich metallic fluids from which diamonds may grow.

The fluxes of carbon from all of these complex and diverse reservoirs have been quantified and modeled. For example, surprising new estimates for the flux of carbon from volcanoes and tectonic regions show the importance of diffuse degassing of carbon dioxide over large regions that are not associated with discrete volcanic eruptions. The deep biosphere has been studied in greater detail than ever before; we now know that the biomass subsisting, in slow motion over millennia—cells gleaning energy from rocks in the subsurface sediments and crust down to a few kilometer depth—amounts to more than 250 times the mass of all humans on Earth. Some of the most astonishing breakthroughs have come from the study of seafloor rocks and fluids. For example, the discovery of amino acids and complex organic compounds in oceanic crust sheds light on the origin of life on Earth through processes linked to “serpentinization”—alteration of seafloor volcanic rocks—and the associated formation of abiotic methane and higher hydrocarbons.

Why it is important that deep carbon research be interdisciplinary crossing over solid Earth, microbiology, and biogeochemistry?

Deep carbon science demonstrates the profound benefits of research that transcends traditional scientific disciplines. Natural phenomena at the varied spatial and temporal scales of Earth cannot be understood without complementary expertise in the physics of materials at extreme conditions, the chemistry of complex fluid–rock interactions, the convective dynamics of subducting plates, the explosive mechanisms of erupting volcanoes, the ancient geochemical origins of life, the diverse metabolic strategies of the deep biosphere, and the epic story of Earth’s coevolving geosphere and biosphere.

No less important are the integration of scientific methodologies. Deep carbon scientists rely on scores of field sites on land and at sea—accessing deep commercial mines, seafloor and continental drilling for subsurface samples, and otherwise studying and sampling remote locations around the globe where rocks reveal aspects of the deep carbon cycle. Equally important are laboratory experiments on chemical reactions of carbon-bearing minerals and fluids at extreme conditions, on the properties of subducting rocks, and on the behavior of deep microbes. Theoretical modeling of geological and biological processes at scales from molecules to the entire globe, coupled with advances in the analysis and visualization of large and growing data resources, provides an essential complement to field and laboratory studies.

What do you think the major breakthroughs in deep carbon science will be over the next ten years?

The next decade will witness a range of new instrumentation and methodologies (including experimentation, modeling, and analysis) becoming mainstream and driving progress in the emerging field of deep carbon science. Deep carbon research, which has brought together a diverse range of geochemists, mineralogists, and biologists, will further evolve as traditional disciplines continue to mix and merge. Analytical capabilities in materials science will drive the characterization of new forms of carbon, motivated by the need for advanced materials for society, as well as for an understanding of planetary interiors. In the field, increasingly miniaturized sensors allow the use of novel platforms such as unmanned aerial systems (known as drones) to sample and analyze high-temperature volcanic gases, which will yield a better understanding of volatile geochemical cycling by plate tectonics and eruption forecasting. The advent of improved high-pressure sampling equipment allows successful capture of complex microbial ecosystems from deep environments, which will pave the way to great progress in quantifying the metabolisms and diversity of deep life.

Data science has an equally important role to play in rationalizing and harvesting enormous datasets and complex multi-dimensional analysis; visualizations and virtual reality platforms that allow collaboration are likely to become routine and established. Advanced modeling capacities will fuel large-scale endeavors to understand the role and form of deep fluids in the mantle, as well as crustal and surface processes, such as the formation of mineral resources, the causes of natural hazards, and the origins of life. Contributing to solutions to pressing societal challenges will be a primary aim, such as developing frameworks to understand carbon sequestration.

In the future, ambitious, integrated monitoring strategies and systems over large areas will characterize the interplay between geological and biological processes in settings such as basins, plate margins, and the seafloor. The development of sophisticated modeling approaches to simulate plate tectonics will be linked with biogeochemical modeling to quantify, in ever more precise detail, the carbon reservoirs and fluxes through deep time to answer big questions related to the development of planetary habitability and the origins of life, both on Earth and possibly other planets.

*The interview was conducted by Senior Editor Dr. Melissa Plail.*

